# Practical Observations on Strangulated Hernia, and Some of the Diseases of the Urinary Organs

**Published:** 1837-04

**Authors:** 


### Art. III.

*Practical Observations on Strangulated Hernia, and some of the Diseases of the Urinary Organs.* By Joseph Parrish, m.d.—*Philadelphia*, 1836. 8vo. pp. 330; with Plates.

Of the various classes into which the writers of medical books may be distributed, according to the motives which have influenced them in becoming authors, there are two which are especially entitled to our attention, and to our respect and gratitude. The first class consists of those stirring spirits, who, while still in the vigour of matured intellect, and floating on the full tide of public favour, find opportunities for the cultivation of literary pursuits, and give to the world, from time to time, the results of their observation and experience on those subjects which have particularly engrossed their attention. That motives of worldly interest and emulation, and a longing after literary fame, should combine in such cases with the purer and more disinterested desire to be useful, will hardly be doubted, and can scarcely be blamed. Whatever the motive, the public are at least the gainers by their labours, and we receive them with pleasure and thankfulness. The second class comprehends those few, unfortunately but necessarily few, whose days have been passed in an incessant career of active practice, and who, towards the evening of life, endeavour to render an equivalent for the public estimation they enjoy, and the honorable distinction which they have acquired amongst their professional brethren, by dedicating their brief space of repose to a review of their past labours, in order that, by collecting the fruits of a long life of experience, they may leave behind them a legacy as valuable to the rising generation as their active and skilful exertions were beneficial to their contemporaries.

We are sorry we cannot include Dr. Parrish amongst this class of authors, without certain limitations. His years, his extensive experience, and, above all, his disinterested motives, well entitle him to be received into such a fellowship; and we can only regret that, with such advantages before him, and with views so evidently philanthropic, he should have fallen so short of the object it was doubtless his intention to accomplish. He appears to have been, through his whole career, a straightforward and pains-taking surgeon, labouring diligently in his vocation, and ministering with the most praise-worthy industry to the bodily sufferings both of the rich and poor: moreover, in the exercise of an extensive practice, he seems to have exhibited an anxiety to embrace every opportunity of increasing his stock of knowledge, both by reading and by personal observation. Nevertheless, we are sorry that, even under all these circumstances, so favorable to improvement, he should have been tempted "to write a book!" However extensive may have been his experience in hernia, and however successful the result of his treatment, we cannot compliment him on having thrown any new light over the pathology of the disease, or of having suggested any improved method of treating it. He repeats and illustrates principles which have been long familiar to every one, but he does nothing more: his book contains a collection of truisms, a detail of symptoms and of modes of treatment familiar to every surgeon, although doubtless the knowledge of these has been acquired and verified by a long course of personal experience.

Dr. Parrish seems to us to show a want of the true spirit of scientific enquiry and of logical inference, and occasionally also a deficiency of anatomical accuracy. This latter defect is the more remarkable in one whose industry is unquestionable, and who must have enjoyed so many opportunities of personal investigation. These remarks we might justify by a reference to almost any part of the volume; but we are not inclined to transcribe its pages, to show how little they contain; neither is it our intention to visit his sins of omission with the severity of criticism, more especially as he seeks to disarm us in his preface, by resigning all pretensions to novelty. But, if we are inclined to pardon the author for not having added more to our stock of knowledge on this important subject of hernia, we cannot be equally lenient towards him with regard to certain parts of his book, where we find him laying down positive rules professedly for the guidance of young surgeons, but which are much more calculated to mislead than to assist them. We allude more particularly to the directions given for the performance of the operations for femoral and inguinal hernia. Dr. Parrish's mistakes are of the more consequence, that, being stated by him with a positiveness arising from evident conviction, they are calculated to become a source of blunder and misconception to the young surgeon.

In speaking of femoral hernia, the author asserts that the edge of Gimbernat's ligament is the cause of stricture. This doctrine is certainly in accordance with a rather favorite notion which formerly prevailed among surgeons, and we believe is still partially current abroad; but the only foundation for Dr. Parrish's belief seems to be the fact, that, when on different occasions he had introduced his finger (in the dead subject) from the groin, through the crural ring into the abdomen, and then carried it inwards towards the pubes, it "hitched" upon Gimbernat's ligament, and was with difficulty withdrawn. No one will dispute the truth of the details of this experiment, but the inference which the author draws from it respecting the seat of stricture is, to say the least, somewhat gratuitous. The direction of the finger, when passed from the exterior to the interior of the abdomen, and then round Gimbernat's ligament, bears not the slightest analogy to the course taken by a femoral hernia; neither does it follow, because the edge of the stricture in question (round which Dr. P. had purposely hitched his finger,) offered an impediment to the withdrawal of the finger, that therefore a femoral hernia, which passes out of the abdomen instead of into it, which then turns upwards, and which moreover does not hitch round Gimbernat's ligament, should be prevented from returning by the same cause. Had it occurred to Dr. Parrish to pass his finger from the abdominal cavity through the crural ring, and then to direct it upwards over Poupart's ligament, thereby simulating the true course of a femoral hernia, he could hardly have fallen into his present mistake; and perhaps, by pursuing this method, he might have become aware of the real cause of stricture, which consists of a little band of fascia or tendon, situated just beneath Poupart's ligament, crossing directly before the neck of the sac, and well known to all those who have carefully dissected the parts in the healthy or the ruptured state. It is, however, but justice to Dr. P. to observe, that, although theoretically wrong, he appears to have been practically right; for, if we understand his directions for liberating the stricture, the edge of the knife is turned in such a manner as would necessarily divide, not Gimbernat's ligament, but the band of fascia above alluded to.

The following passage occurs in the description of the operation given by our author:

"The situation of the neighbouring vessels also offers an additional reason for deliberate and careful proceeding. It will be recollected that the great femoral artery and vein are contained in the same sheath which envelopes the hernia, and the epigastric artery should also be borne in mind. The last-mentioned vessel arises from the external iliac, just as it passes under Poupart's ligament, where it takes the name of inguinal artery: hence its origin is very near to the outside of the reflected edge of Poupart's ligament, called Gimbernat's ligament, which is the seat of stricture: a very slight division of the ligament outwards would separate the artery at its origin. In dividing the stricture, it is important to have the finger as a guide, and by all means to effect the division upwards, and rather inward." (P. 34.)

We must acknowledge ourselves fairly mystified by this passage. We cannot understand how, by any contrivance whatever, it can be possible to divide the epigastric artery *near its origin* from the iliac, while cutting against the edge of Gimbernat's ligament; the neck of the sac itself and the femoral vein being necessarily interposed between the knife and the seat of the dreaded mischief. We may also be allowed to doubt whether the operator does really divide Gimbernat's ligament by directing the edge of the knife upwards and rather inwards. That Dr. P. does not confound the epigastric with the occasional variety which occurs in the origin and course of the obturator vessel is evident, as he afterwards mentions that variety, and states that on one occasion he distinctly felt the obturator artery beating against his finger immediately over the seat of stricture. In another part of the book, he speaks of the vicinity of the spermatic cord as likely to excite the apprehensions of the young operator, while dividing the stricture in femoral hernia. Now, (setting aside the fact that crural hernia occurs but rarely in the male,) we must say that the young surgeon must have studied his anatomy to little purpose, or he need be under no great apprehension of bleeding his patient to death by dividing the epigastric artery, or emasculating him by cutting the cord.

Near the beginning of the volume, Dr. Parrish alludes to the method of dividing the stricture outside the sac, and then returning the contents without opening the sac itself; a plan which was recommended by some of the earlier surgeons, and has lately been revived and successfully practised by Mr. Key. This plan he decidedly deprecates, and states his fixed resolution never to adopt it under any circumstances. He devotes some pages to this subject; but the reasons which he gives for making it an invariable rule to bring the contents of the hernia into view, amount merely to a repetition of the well-known fact, that there are some circumstances under which it would be more unsafe to return an intestine to the abdomen than to leave it in the sac. He forgets that the possibility of such contingencies as gangrene, internal stricture, &c. would operate equally in deterring us from reducing a hernia under any circumstances, or employing the remedial means usually adopted for the purpose of avoiding the necessity of an operation. He seems hardly to be aware of the object and intention which the surgeon has in view by dividing the stricture external to the sac; namely, that he may employ the taxis under a condition of parts which he has rendered more favorable to its successful application, in preference to subjecting the patient to the risk of peritoneal inflammation by opening the abdominal cavity.

As, however, the excellent memoir in which Mr. Key has explained and advocated this method of reducing hernia must be known to most of our readers, (although our author does not appear to have read it,) we do not think it necessary to enter into any controversy on the question. An assertion which Dr. P. makes, that wounds into the serous cavity of the abdomen are not liable to be followed by peritonitis, will hardly be corroborated by his professional brethren. Neither are we warranted in drawing an analogy between the accidental wound of a healthy serous membrane, and an incision made through a portion of the peritoneum already inflamed, together with the exposure of a morbidly sensitive intestine to the influence of the air and the manipulation of the surgeon. In another part of the book we find the details of a case in which our author attempted, although unsuccessfully, to relieve the stricture without opening the sac. It seems, however, to have been an unfortunate instance in which to make trial of that method, as strangulation had existed during four days, and, on exposing the contents of the hernia, the omentum was found in a state of complete gangrene.

We shall devote the remainder of this article to the notice and arrangement of certain facts, as we find them scattered in the detail of a vast number of cases, which constitute the chief bulk of the volume. We heartily wish that the author himself had saved us this trouble, and that he had shown himself more capable of appreciating the useful application of his own labours and great experience; from which we certainly had a right to expect some valuable principles, some practical observations, or new suggestions, applicable both to the diagnosis and the treatment in obscure and difficult cases of this important disease. We shall do Dr. Parrish all the justice in our power, by quoting his own words whenever it is practicable.

The practice of giving large doses of opium previous to an operation for hernia is not very general among our own surgeons, but was invariably adopted by Dr. Parrish, and apparently with beneficial results. He thus states his belief in its efficacy:

"When the operation is concluded upon, it is my uniform practice, as in most other operations, to give an opiate either by the mouth or rectum. I am aware that the general application of this practice is objected to by some men of high character: it is said that opium is a stimulant, and tends to excite the system, produces fever, &c. This fear, I believe, is grounded in theory rather than in practice. I give opium to prevent fever, and believe the practice to be not only successful but rational. The calming influence generally produced by this article tends to lessen the pain of surgical operations, and the shock which they occasion; and hence it assists in mitigating one of the great sources of subsequent reaction and fever. I have never seen any other effect produced by it, although I have employed it very generally in my surgical practice." (P. 28.)

The dose of opium usually administered, as far as we can gather from the history of cases, was from one to three grains. He appears also to have considered it as a valuable article in the treatment for the reduction of strangulated hernia, and far preferable to the depleting and depressing measures generally employed, such as the warm bath, bloodletting, and tobacco: since these exhaust the powers of the patient, rendering him less capable of undergoing the shock of an operation, should such an alternative become inevitable; whereas, opium has a directly contrary effect. Indeed, the success which generally attended the operations of Dr. P. appears mainly attributable to his having early recourse to the knife without suffering the vital energies of his patient to be destroyed by previous depressing remedies. He considers sixty drops of laudanum, exhibited by the rectum, as fully equal to thirty given by the mouth. The moderate use of the lancet, followed up by a very full dose of opium, seems to have been the favorite means adopted by Dr. P. for the reduction of hernia.

At page 88 we find the details of a rather interesting case, in which a femoral hernia, after having resisted all the ordinary means for reduction, yielded at length to the remedies of a quack doctor. The subject of the case was a woman, aged fifty, in whom an old crural rupture had suddenly become strangulated. The usual succession of symptoms came on, but marked with peculiar violence; she obstinately refused to be relieved by operation; fecal vomiting supervened; and, on the evening of the third day, Dr. P. left her, as he considered, in *articulo mortis*. We give the sequel in his own words:

"On calling the next morning to see the patient, I found her still alive, and that she had called in a black man, celebrated in the Neck, (the low country south of the city,) as 'a curer of ruptures' both in men and in cattle. I remained, being somewhat curious to watch his proceedings. The hernial tumour he had covered with a poultice of bruised herbs,—the leaves, so far as I could judge by the smell, of stramonium,—and he was preparing an infusion of herbs to be used as an injection. This infusion was evidently of senna leaves. The injection he proposed to administer every fifteen minutes, by means of a very large and very powerful syringe. He spoke confidently of the successful result of the case. I saw the patient again on the following morning, and, to my astonishment, found her in a tolerably comfortable condition! The hernia was reduced; all the alarming symptoms, under which she had laboured on the preceding day, were gone; and, though extremely weak, she was evidently in a fair way for recovery! I learned that after continuing the injections for nearly two hours, there occurred a copious evacuation from the bowels, of a number of hard balls; and that then suddenly the tumour had disappeared with a gurgling noise. These balls had been preserved for my inspection: they were formed of hard, dark-coloured feces of different sizes, from that of a pea to that of a pistol ball, or even larger. The patient continued daily to amend, and, at the termination of ten days from the reduction of the hernia, was seen by me sweeping off her door! September 19, 1835. I saw her this day. She enjoys excellent health; and, so far as I am able to say without an actual examination, is radically cured of her hernia." (P. 91.)

The use of stramonium, although a powerful sedative and narcotic, has not, we believe, been adopted as an application in cases of hernia; but we gather from the details of a subsequent case that the hint afforded by the successful operator in the foregoing one, was not thrown away upon Dr. Parrish, as we find him diligently covering the abdomen with the leaves of the plant, and administering injections of senna. Although we are far from denying the beneficial effects of stramonium, and should much wish to give it a fair trial ourselves; yet we cannot quite agree with the Doctor's observation on the rationale of the treatment, when he says,

"Every practitioner is familiar with the dilating power of the extract of stramonium over the iris, and in this case the poultice certainly did appear to dilate the abdominal ring." (P. 70.)

We are not aware whether Dr. P. was generally in the habit of exhibiting injections with a view to effect the return of the intestine previously to having recourse to an operation, and he makes no mention of them amongst his means for reduction. We regret not having the recorded benefit of his experience on this point, as we believe that the use of emollient or purgative enemata, introduced high up into the intestine by means of a long pipe, may be rendered of powerful avail; more especially in cases of old hernia, where there is reason to suppose that an habitually constipated state of the bowels existed previously to the strangulation.

The sudden evacuation of a large quantity of feculent matter and flatus, which often follows the administration of these remedies, and the consequent diminished volume of the contents of the abdominal cavity, together with the peristaltic action which is probably set up in the intestinal canal, are calculated to produce the happiest effects in the reduction of a rupture. The profession is much indebted to Dr. O'Beirne, of Dublin, for his valuable suggestions on this subject, in his interesting and original work on Defæcation.

We are surprised that Dr. Parrish does not allude farther to the use of injections, as he seems fully aware that the large intestines are frequently overloaded, and warns the practitioner not to imagine, from the casual occurrence of an evacuation during the symptoms of strangulation, "that the intestinal canal is therefore necessarily free from the stomach to the anus." A similar delusion, he says, may be produced on the mind of the surgeon by the return of the clyster tinged with the fecal colour and possessing the fecal odour. We think his experience might have warranted his going much farther in his statement, and informing his readers that it is not very uncommon to obtain copious feculent dejections from the large intestines under the use of enemata, while at the same time a portion of the ileum or jejunum may be retained in a state of firm strangulation.

Almost every surgeon of much experience will have seen cases of hernia where the symptoms of strangulation continued after the bowel had apparently been returned by the taxis: or, again, it sometimes happens that an operation, although promising and satisfactory to the surgeon, nevertheless fails in producing the expected result. The vomiting remains unchecked, the constipation is not relieved, the pain and tension of the abdomen become increased, and the patient dies without any cessation of the original symptoms. In these cases of perplexing difficulty, Dr. P. places great reliance on the continual administration of calomel in small but very frequent doses.

"A train of the most alarming symptoms may continue for days after reduction, and yet may yield to appropriate medical treatment. The remedy upon which I have placed chief reliance in these cases is mercury, introduced into the system in extremely minute portions. Calomel, in the dose of one-quarter or one-sixth of a grain, given every one or two hours, is the form I would recommend." . . . . "I shall not attempt to discuss at large the *modus operandi* of this remedy. It is well known that calomel, even in very small portions, has the power of correcting functional derangement of the liver and of exciting the flow of healthy bile. That bile of this description is the most natural excitant of the bowels, and is admirably calculated to promote steady and healthful peristaltic action, it is presumed will be generally admitted. The introduction of calomel in such minute portions as not to offend the stomach, or to produce any constitutional irritation, is peculiarly appropriate in diseases where the stomach is irritable, and in none more so than in strangulated hernia. Large doses of calomel excite the liver and alimentary canal to increased action, and thus transcend that medium which it is so desirable to maintain: hence, while powerful doses are obviously injurious in cases of recent strangulation, the accumulated effect of minute portions frequently repeated may be attained; while at the same time the irritability of the stomach is allayed." (P. 64.)

The cases are given in illustration of these remarks. In the first, the most violent symptoms of strangulation supervened after the hernia had been returned by the taxis. Stercoraceous vomiting, accompanied by a tympanitic state of abdomen and obstinate constipation, continued for six days, when copious evacuations were at length obtained from the bowels, and the recovery became rapid. In a second similar case, the bowels were not relieved until ten days from the commencement of the original attack: the vomiting, which was at first incessant, became afterwards checked. In the third case the hernia was returned by operation, after which eight days elapsed before evacuations could be obtained; and, during this time, the patient manifested much of the ordinary symptoms which indicate a fatal termination from strangulated intestine. Vomiting, however, does not appear to have been present. In these three instances calomel was constantly administered in divided doses; but, as we likewise learn from the reports that the patients were vigorously plied with purgatives of various descriptions, exhibited by the mouth and injected into the rectum by means of a long elastic tube, it is rather difficult to give a decided opinion as to the specific effect of the mercury, of the salutary operation of which we have already given the author's explanation. In the first case reported, it could not have been very efficacious, as the patient appears to have thrown up all the ingesta admitted into his stomach, together with, occasionally, the contents of the small intestines. In the last two cases the calomel was probably more effective, as the gums of both the patients exhibited the usual appearances produced by that medicine. Dr. Parrish makes no particular mention of instances of this kind accompanied by a fatal result: we have been unfortunate enough to witness the post-mortem examination of some few, in which the inflammation originally produced at the strictured part had slowly extended along the course of the intestine, sometimes accompanied by general or partial peritonitis. A want of vital power, necessary to the restoration of the injured parts, generally seems to prevail in these cases; and the surgeon thus finds himself thrown on to the horns of a dilemma, having to combat the progress of inflammation on the one hand, and to sustain the sinking energies of his patient on the other.

From the chapters dedicated to the operation, we select some remarks on the treatment to be adopted under various states and conditions of the contents of the sac.

When the intestine is found to have contracted adhesions to the surface of the sac, and more particularly about its neck, the author's practice has constantly been to separate these, and at all events to return the gut. In such cases there is often a layer of fresh adhesive matter coating the intestine: this he removes with the handle of his scalpel. Indeed, whenever the intestine is coated with lymph in consequence of inflammation, even where no adhesions to the sac are present, he considers it necessary to peel off the adventitious covering before restoring the gut to the abdomen. This we should have considered rather a work of supererogation, if not a meddlesome interference with the operations of nature; but, as our author cites several cases in which the practice was adopted, and the result successful, it may be useful to know that the parts will bear this denudation, and the patient recover after what would be considered by some surgeons as unnecessary, if not a hazardous proceeding.

We pass over the section on Gangrene, in order to advert to the subject of entero-epiplocele. The opinions of medical men are still somewhat at variance respecting the best treatment to be adopted with regard to the omentum. When the omentum contained in the sac is moderate in quantity, and has recently descended along with the intestine, there can be no doubt respecting the propriety of returning it, and little difficulty will probably be experienced in so doing: but, on the other hand, a source of doubt and perplexity always presents itself when a surgeon finds the greater part of a hernial sac occupied by a large mass of omentum, which, from its alteration in form or its morbid condition, he either cannot or ought not to return into the abdominal cavity. He then feels puzzled to decide how he is to dispose of it with most advantage, or rather with least danger to his patient. The conditions of protruded omentum which thus become a source of difficulty to the operator are two: 1st, in old hernia, where the omentum has long lain within the sac, and has become hardened, conglomerated, and moulded to the cavity; 2d, where it is gangrenous, or in a state approaching to gangrene.

Dr. Parrish expresses himself in the following manner with regard to the first condition, which he, in rather metaphorical language, designates "expatriated omentum."

"Some readers," he says, "may smile at this term; but perhaps they may be convinced that it conveys a brief but just illustration of the condition of the parts. It is possible for a man to absent himself for so long a period from his native country, that his early associations may be completely dissevered. He may acquire new views, he may cultivate other affections, and may become estranged from the land which gave him birth. In the course of events, such an alien from his country may return as an enemy, clothed in hostile array. So it is with the omentum; a portion of this structure may be separated for so many years from the cavity of the abdomen, that it may entirely lose its native character. Instead of a soft yielding apron of fat, destined to spread over the delicate bowels, it may become converted into a solid mass, bearing no resemblance to its original structure, and totally unfitted for the performance of its appropriate functions. It is expatriated, and has become an alien from its native home. If in this condition it be forcibly returned within the cavity from which it originally escaped, it may act as an extraneous body, and may prove an agent of discord, danger, and death." . . . "The question then arises, what is to be done with expatriated omentum? If a small portion present itself, and can be conveniently cut off, no danger need be apprehended from hemorrhage; but, when a large mass is encountered, it has generally been my practice to allow it to remain undisturbed." (P. 123 and 145.)

With regard to the propriety of returning "expatriated omentum," the question is generally set at rest by the impossibility of effecting its reduction; for, in most cases, either the adhesions which it has formed will prevent the restoration of the stranger to his long forsaken home, or else, like Æsop's weasel in the granary, it has become so bulky and unwieldy as no longer to allow of its re-entrance through the same opening by which it originally escaped. The question, therefore, upon which nine times out of ten the surgeon will have to decide, is whether, having reduced the intestine, he shall leave the omentum in the sac or cut it off; and here we cannot help observing, that the alternative which Dr. P. has adopted in his practice appears to us both unscientific and erroneous. If he considers that it is safe, or rather that it is expedient, to leave a large mass of omentum in the sac, why should he resolve upon removing a small quantity, which can produce no farther inconvenience by being suffered to remain in a situation where probably it has existed for years. No one, we conceive, would be inclined to meddle with "expatriated omentum" if it could be avoided; and it is only when the quantity is so large that it protrudes through the external wound, and prevents the edges from becoming approximated, that we should ever be disposed to take the propriety of its excision into consideration. Yet Dr. P. decides that a small portion shall be removed, because it can be done conveniently. A broken arm may be cut off with great convenience to the surgeon, yet such treatment would hardly be met by a reciprocity of feeling on the part of the patient.

The probability of hemorrhage may indeed be a reason for declining to excise a large mass of omentum, (although we should not, in such cases, contemplate any risk of internal bleeding, as it is most unlikely to be drawn up into the abdomen;) but the exemption from this danger can be no reason for determining on the removal of a small portion. Five cases of operation are given, where intestine had become strangulated in a sac containing "expatriated omentum." In three of these cases the omentum was removed, and the patients recovered: the history, however, is incomplete as regards the information for which they are detailed, as no mention is made as to the quantity of omentum, or whether it had contracted adhesions; but, as they are given in illustration of the author's views, we suppose the "expatriated" mass to have been inconsiderable. In the two remaining instances, where the omentum was considerable in quantity, it was suffered to remain. They both terminated fatally, but we should observe that the patients appeared to be in a hopeless state at the time of the operation.

When the omentum is found to be in a gangrenous condition, the treatment recommended by Dr. Parrish is as follows:

"Believing that the excision of a large mass of omentum is attended with risk by any method, I have pursued the practice of leaving the mortified portion in the wound, relying upon the efforts of nature to effect its separation from the sound parts. This process may be assisted by the gradual yet very gentle pressure of a ligature around the root of the diseased mass, in such a manner that the patient may at any moment unloose it, if he should feel pain or sickness." (P. 159.)

One case is given in which this treatment was adopted. The operation was performed on the fourth day after strangulation had taken place. The sac contained about eight ounces of omentum partially sphacelated. After returning the intestine, the lower part of the wound was closed by sutures, and the omentum left protruding above. The gangrenous portion speedily separated; a ligature was then applied around the sound part, which still remained exposed. Its entire removal was subsequently effected by this means, and the patient recovered. Another case of entero-epiplocele is related, where the intestine was returned without operation; the omentum which remained in the sac became gangrenous, and was eventually discharged by spontaneous abscess. The patient perfectly recovered.

The history of Dr. Parrish's cases, however, and the result of our own experience, would much incline us to avoid any unnecessary interference with protruded omentum, and to place our chief reliance in the spontaneous operation of nature for effecting a favorable termination. The instances in which a removal of any portion might be deemed expedient, are those where adhesion had taken place between the omentum and the neck of the sac, so as in some degree to shut out its direct communication with the abdomen.

We must here close our review of Dr. Parrish's book. We have endeavoured, at considerable pains to ourselves, to cull from it whatever we considered might afford practical information to the surgeon; and it has more especially been our aim to render the recorded experience in difficult cases subservient to the establishment of some fixed principles by which his treatment may be regulated. The imperfect arrangement and the desultory style of the author have tended greatly to impair the general utility of the work; and we can only hope that he has rendered his experience more available to his patients than it will ever be to his readers. Dr. Parrish is, doubtless, an excellent man, and a good practical surgeon; but his forte is not in writing books.

